# Aggressive behaviour of anti-vaxxers and their toxic replies in English and Japanese

**DOI:** 10.1057/s41599-022-01245-x

**Published:** 2022-07-05

**Authors:** Kunihiro Miyazaki, Takayuki Uchiba, Kenji Tanaka, Kazutoshi Sasahara

**Affiliations:** 1 The University of Tokyo, Tokyo, Japan; 2 Sugakubunka Co., Ltd., Tokyo, Japan; 3 Tokyo Institute of Technology, Tokyo, Japan

**Keywords:** Social policy, Complex networks, Cultural and media studies

## Abstract

The anti-vaccine movement has gained traction in many countries since the COVID-19 pandemic began. However, their aggressive behaviour through replies on Twitter—a form of directed messaging that can be sent beyond follow-follower relationships—is less understood, and even less is known about the language use differences of this behaviour. We conducted a comparative study of anti-vaxxers’ aggressive behaviours by analysing a longitudinal dataset of COVID-19 tweets in English and Japanese. We found two common features across these languages. First, anti-vaxxers most actively transmit targeted messages or replies to users with different beliefs, especially to neutral accounts, with significantly toxic and negative language, and these replies are often directed to posts about vaccine operations. Second, influential users with many followers and verified accounts are more likely to receive the most toxic replies from the anti-vaxxers. However, pro-vaccine accounts with a few followers receive highly toxic replies in English, which is different from the Japanese case. These results provide insights into both language-dependent and independent countermeasures against anti-vaxxers’ aggressive behaviour.

## Introduction

The COVID-19 pandemic began at the end of December 2019 and spread rapidly worldwide, affecting both the global economy and health. The pandemic also caused the overabundance and spread of misinformation related to COVID-19, such as incorrect treatments, conspiracy theories, and pseudoscience about vaccines (Agley and Xiao, 2021), which induced secondary damage to society. In particular, the vaccine hesitancy caused by anti-vaccine advocates, or anti-vaxxers, who spread misinformation and anxiety-provoking information, is an urgent social problem, as it may delay or hinder the widespread of vaccination (Burki, 2019) and the achievement of herd immunity necessary for a post-pandemic world (Fontanet and Cauchemez, 2020). The World Health Organization (WHO) has called such a flood of mis/disinformation under the pandemic an ‘infodemic’^1^. To solve this problem, a scientific understanding of online anti-vaccine behaviour and effective countermeasures across countries are required.

Much research has focused on the social media ecology of anti-vaxxers, or anti-vaccine advocates; their thoughts and claim contents (Brennen et al., 2020; Kata, 2012); behavioural patterns, emotions, topics, and positions in the social networks (Germani and Biller-Andorno, 2021); changes in their attitudes over time (Mitra et al., 2016); temporal patterns of how fake information spreads (Gunaratne et al., 2019); and motivations for sharing misinformation (Apuke and Omar, 2021). Although many attempts have been made in these directions, we have yet to understand the most direct messaging form for anti-vaxxers’ beliefs on social media, that is, reply behaviour. Unlike other forms of messaging behaviours (e.g., posts, shares), replies can be sent beyond the boundaries of follow-follower relationships, thereby gaining many impressions^2^ by being viewed by the targets and their followers. Especially on vaccine topics, users are often inside ‘echo chambers’, only seeing beliefs they want to see (Sasahara et al., 2020), but in reality, a reply can reach anyone regardless of the follow-follower networks (Choi et al., 2020)^3^. Furthermore, most of the existing studies on anti-vaxxers’ behaviours have analysed English data, and it is unclear whether the findings from these studies are valid across different languages.

In this paper, we empirically examine the characteristics of anti-vaxxers’ reply behaviours in the context of COVID-19 infodemic by analysing a longitudinal dataset of tweets in English and Japanese (see Data collection). The ratio of replies among all tweets turned out to be considerably small; nevertheless, the reply behaviours of anti-vaxxers are indispensable for research because the recipients can be seriously affected by the negative replies from an unknown user outside of their follow-follower networks. We found that in both languages, anti-vaxxers most frequently sent replies to clusters with different beliefs, especially to neutral accounts, and the content of their replies was significantly toxic and emotional. Furthermore, the most-targeted users were conspicuous accounts with large numbers of followers, including accounts related to healthcare or representing scientists, policymakers, media figures, or outlets. Based on the findings, we finally discuss possible countermeasures in multilingual settings, which can be useful for pro-vaxxers, fact-checkers, and platformers for creating guidelines and automated systems that detect harmful content, including mis/disinformation about vaccines.

## Methods

### Data collection

We continuously used Twitter Search API to collect a comprehensive dataset of COVID-19-related tweets from February to December 2020. The query terms for this search include ‘corona virus’, ‘coronavirus’, ‘COVID19’, ‘2019-nCoV’, ‘SARS-CoV-2’, and ‘wuhanpneumonia’ and their counterparts in Japanese. We then extracted tweets that contained any of the following vaccine-related words: ‘vaccine’, ‘vax’, and ‘vaccination’, and their counterparts in Japanese, and then retained them for analysis. The resulting volume of English tweets was 8,579,728, of which 6,879,713 (80.2%) were retweets (RT) and 293,946 (3.43%) were replies (RP). The number of unique users was 2,799,034. For Japanese tweets, we obtained 1,952,376 tweets, of which 1,591,410 (81.5%) were RTs, 51,685 (2.64%) were RPs, and the number of unique users was 576,894. Note that we did not include ‘vaccine’ in the search query for the above-mentioned crawling, because our focus is on vaccine-related tweets in the context of the COVID-19 infodemic. Therefore, we first collected the COVID-19 tweets and then filtered tweets with the above vaccine-related words, rather than the other way around. At the time of the start of the search, the WHO had not declared a pandemic and therefore we did not include ‘pandemic’ in the query term in the succeeding search for data consistency.

### Networks of anti-vaxxers and other groups

We employed the RT network-based clustering to classify users according to their vaccine stance. RT network clustering detects users with similar stances by applying network clustering to an RT network (Conover et al., 2011, Fortunato, 2010). We used RT network clustering in our study for three reasons. First, on the topic of vaccines, research has illustrated that a network community can be easily divided by their stance on vaccines and emerges echo chambers (Cossard et al., 2020; Gunaratne et al., 2019), which led us to believe that we could get anti-vaccine clusters using this method. Second, RT network clustering can automatically reveal clusters of like-minded users without imposing any thresholds for classification. Last, it does not present the problem with an arbitrariness that differentiating pro-vax from anti-vax hashtags does.

To construct the RT network, using all data from February to December 2020, we created an edge between users with more than two RTs (including mutual RTs). As a result, the meaning of the endorsement was more robustly incorporated into the edges (Garimella et al., 2018). After creating the network, we applied *k*-core decomposition (*k* = 3) to exclude users with only weak connections to the primary discussions (Alvarez-Hamelin et al., 2006). Next, the Louvain method was used to cluster anti-vaccine users and other groups (Blondel et al., 2008). Owing to the constraints RT ≥ 2 and *k*-core = 3, the number of users was reduced to only those participating in the discussion about vaccines.

**Fig. 1:**
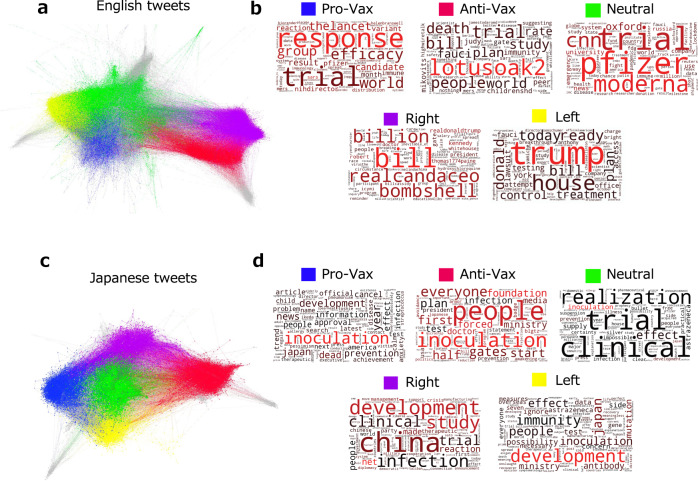
RT network. RT network (**a**) for English users and the corresponding word clouds (**b**) and Japanese counterparts (**c**, **d**). In **a**, the number of nodes is 47,135, and the number of edges is 241,370, while in **c**, the number of nodes is 12,017, and the number of edges is 62,132. Nodes represent users and links represent retweets, and colours correspond to clusters. The five biggest clusters were found in both networks: in **a**, Pro-Vax (9.17%), Left (Anti-Trump) (16.31%), Neutral (24.84%), Anti-Vax (12.23%), and Right (Pro-Trump) (12.18%); in **c**, Pro-Vax (18.6%), Left (16.02%) Anti-Vax (19.5%), Neutral (25.42%), and Right (10.86%). In the word clouds **b** and **d**, font size corresponds to keyword frequency, and font colour corresponds to their Tf-Idf values (i.e., word importance). In **d**, Japanese keywords are translated into English.

We found the five clusters by the method mentioned above and named each cluster by looking at their retweeted texts (see the word clouds in Fig. 1 and raw texts (SI)) and the representative accounts that were most retweeted.

### Measurement of toxicity

To measure the degree of attack conveyed by tweets toward others on Twitter, we employed Google’s Perspective API^4^, a popular tool widely used for online abuse and harassment research (Hua et al., 2020a, b; Wu and Resnick, 2021). The perspective API allows users to measure the toxicity of a text in English on a scale from 0 to 1. For measuring the toxicity from the text in Japanese, we needed to translate it to English, as the perspective API is only available in English. To this end, we used the Google Translate translator API^5^ (Samoilenko et al., 2017).

### Measurement of emotions

To evaluate the emotions of replies, we adopted an approach of counting the words registered in the sentiment dictionary. For the positive and negative emotions, we used the LIWC 2015 dictionary (Pennebaker et al., 2015). As for the level of arousal and valence, we used the dictionary of Warriner et al. (2013). We counted the number of words per tweet with scores above the median for each metric in the dictionary because the dictionary also contains words with low scores. In measuring emotions, we translated Japanese tweets into English using the Google Translate translator API, the same as for measuring toxicity.

## Results

### Communities related to vaccine discourse

We constructed the retweet (RT) networks from the COVID-19 tweets to identify anti-vaxxers and other groups (see Methods). The resulting networks are illustrated in Fig. 1a for English and 1c for Japanese. Figure 1b, d illustrate popular words used in each group.

We found five clusters or groups in English tweets (Fig. 1a). Although these groups commonly used factual words related to COVID-19 and vaccines (‘trial’, ‘response’), they also used different keywords specific to each group (Fig. 1b). One group paid much attention to the efficacy and the evidence of vaccines (‘efficacy’, ‘the lancet’), suggesting the Pro-Vax group. Another group focused on conspiracy theories and criticism of the government (‘bill’, ‘fauci’^6^^,^^7^), suggesting the Anti-Vax cluster; especially, a large portion of conspiracies consisted of Bill Gates, one of the biggest proponents of vaccination^8^. Another group that mentioned the topics of Bill Gates’s investment in vaccines and some political names (‘bill’, ‘billion’, ‘kennedy’, ‘realdonaldtrump’) seemed to be a political right cluster, whereas the one that contained words for politics and government (‘trump’, ‘house’) seemed to be a politically left cluster. In addition, we identified one that contained vaccine makers, universities, and news media outlets (‘cnn’, ‘oxford’, ‘pfizer’, ‘moderna’) as a neutral cluster. We further confirmed the above observations by checking each group’s retweeted accounts and tweets for each group (see Supplementary Information (SI)). While in most clusters, the popular accounts included various users, such as politicians, journalists, doctors, anonymous influencers, and organisations (WHO, the White House), the neutral cluster was mainly occupied by news media, such as CNN and Reuters (see SI).

The comparable five clusters with topic structures emerge in the Japanese dataset (Fig. 1c): the pro- and anti-vaxxers, the political Left and Right clusters, and the Neutral cluster. Besides factual words (‘inoculation’, ‘development’), the Pro-Vax group used words related to the efficacy of vaccinations (‘prevention’, ‘effect’), whereas the Anti-Vax group used words related to conspiracy theories and criticism of the government (‘gates’, ‘ministry’^9^). In the political clusters, the Right cluster criticised China (‘china’), whereas the Left group criticised the government (‘ministry’). In addition to Japanese news media, vaccine makers’ accounts (‘astrazeneca’) and words related to the possibility of vaccination (‘realisation’, ‘clinical trial’) were found in the neutral group. Thus, the vaccine information ecosystems demonstrate striking similarities in English and Japanese, although they have different vaccine policies and political backgrounds. This structural and topical resemblance suggests that vaccination is a common political matter across countries (Sharun and Dhama, 2021), and was perhaps weaponised during the COVID-19 infodemic (Broniatowski et al., 2018; Jamison, 2020). To vaccinate or not is a matter of intersection between personal freedom and public health policy (DiResta, 2018). Therefore, the vaccine topic may intensify conflicts between groups with different ideologies and beliefs, which will be further discussed later.

### Active reply by anti-vaxxers

**Fig. 2:**
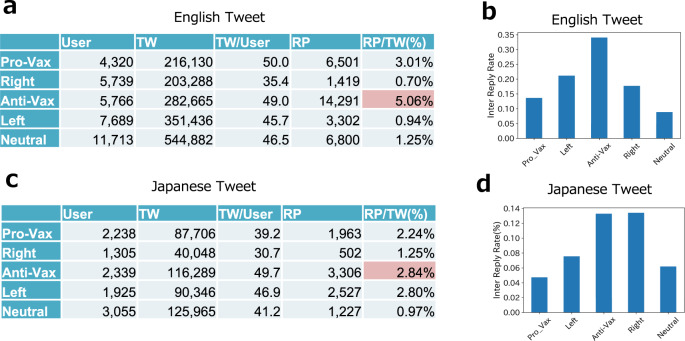
Tweet and reply activities between clusters. **a** Reply activities of each cluster in English tweets. Here, TW: tweets. RP: replies. TW includes normal tweets, RP, and RT. Reply rate (RP/TW) was significantly higher in the Anti-Vax cluster (*χ*^2^ test: *p* < 0.001, illustrated in rad shades); **b** Ratio of inter-cluster replies per all replies from each cluster in English tweets; **c** and **d** are the Japanese counterparts.

After identifying the groups, we analysed how actively anti-vaxxers targeted other groups using replies. Figure 2a, c illustrate that both in English and Japanese, anti-vaxxers were the most active in reply behaviour. Looking at the reply frequencies in inner-cluster (i.e., replies to the same cluster) and inter-cluster (i.e., replies to the other clusters) conditions, we find two similarities in both languages. While most replies were directed towards the same cluster (inner-cluster), the Anti-Vax groups sent the largest number of inter-replies (n.b., the Right cluster is comparable in Japanese). Thus, anti-vaxxers are supposed to be more enthusiastic about reaching out to people with other beliefs. In contrast, pro-vaxxers sent out direct messages to external clusters at a lower frequency according to the inter-reply rate, although they should have contributed to disseminating the correct knowledge about the COVID-19 vaccination.

### Reply targets of anti-vaxxers

**Fig. 3:**
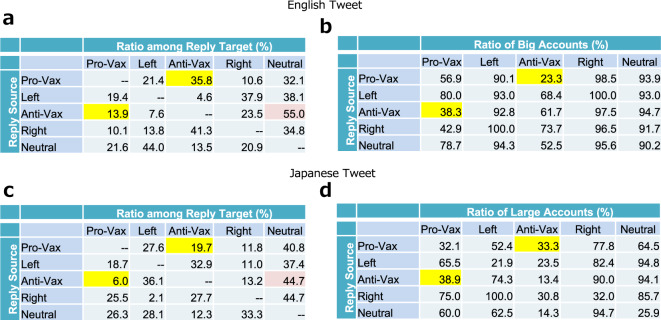
Ratio of targeted clusters and popular accounts in inter-cluster replies. **a** Ratio of inter-cluster replies by source and target pairs in English; **b** Ratio of popular accounts (with the number of followers ≥10,000) in each targeted cluster. **c** and **d** are Japanese counterparts of **a** and **b**, respectively.

Next, we examined the main targets of anti-vaxxers (Fig. 3a, d). Although all the groups were mainly targeting the Neutral group, the Anti-Vax groups had this tendency more apparent than others (shaded in red) in both languages (especially in English). This result is consistent with the findings of previous research that anti-vaxxers are more entangled with neutral groups than pro-vaxxers and thus, successful in their reach (Johnson et al., 2020). On the contrary, the rate of replies from Anti-Vax to Pro-Vax is lower than that of their opposite counterparts in both languages (shaded in yellow). This asymmetry in reply frequency suggests that the Anti-Vax group tends to neglect the Pro-Vax group.

Furthermore, we found that the reply targets have large numbers of followers, and the median scores of followers of reply receivers are much larger than those of reply senders (see SI). To quantify the tendency of replies toward influential accounts, we calculated the percentage of replies directed to accounts with more than 10,000 followers (Fig. 3c, f)^10^. We found that most of these neutral accounts have numerous followers, and the neutral accounts that received the most replies from anti-vaxxers were media and politicians’ accounts. Contrarily, the percentage of replies from anti-vaxxer to pro-vaxxer groups was considerably small (shaded in yellow).

### Highly toxic replies by anti-vaxxers

**Fig. 4:**
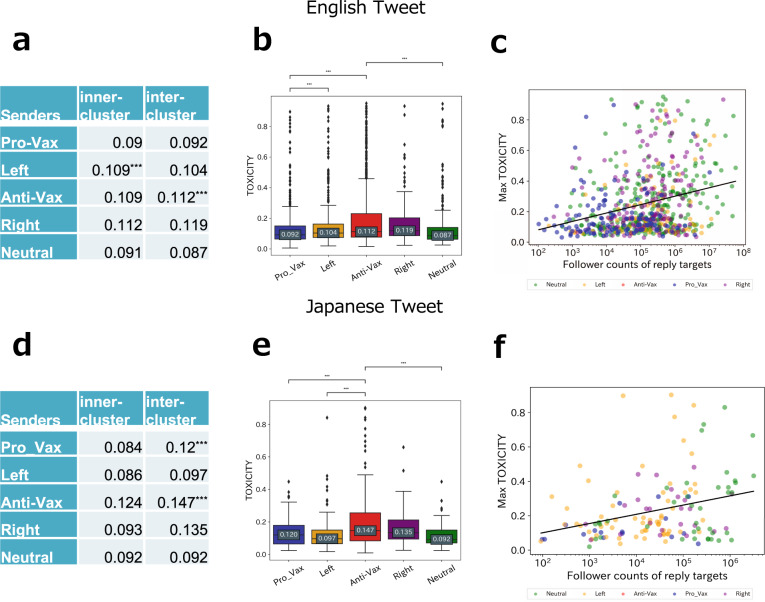
Toxicity scores. **a** Median toxicity scores of inner-cluster and inter-cluster replies from each cluster. The Anti-Vax's inter-cluster replies have a significantly higher toxicity score than its inner-cluster replies. The Left's inner-cluster replies have a significantly higher toxicity score than its inter-cluster replies. Both have *p* < 0.001 by Mann–Whitney *U*-test with Bonferroni correction. **b** Boxplots represent the toxicity of the inter-cluster replies from each cluster. Each data point indicates the toxicity of a reply. Each median score is annotated in the boxes. Replies from the Anti-Vax cluster were significantly more toxic than ones from the Pro-Vax and Neutral clusters in both languages (^***^p < 0.001 by Mann–Whitney *U*-test with a Bonferroni correction). **c** The maximum toxicities that one received from Anti-Vax users. Each data point in the figure is the users who received the reply. The *x*-axis is the number of followers of the users, and the *y*-axis is the toxicity of the replies received. The correlation coefficients are **d**: 0.02 (*p* = 0.601), **e**: 0.198 (*p* < 0.001), **h**: 0.096 (*p* = 0.254), and 0.268 (*p* < 0.005). **d**, **e**, and **f** are Japanese counterparts of **a**, **b**, and **c**, respectively.

To characterise the nature of anti-vaxxers’ inter-replies (i.e., replies to other groups), we measured the toxicity of languages used in replies (see Methods) and tested the differences between inter- and inner-replies in each group (Fig. 4a, d). We found that the inter-cluster replies were significantly higher than the inner-cluster replies in both languages.

The toxicity of the inter-reply is compared between clusters in Fig. 4b, e. This reveals that the toxicity of the Anti-Vax’s inter-reply is higher than the other groups, especially significantly higher than those of the Pro-Vax and Neutral groups in both English and Japanese^11^. Next, we examined who was more likely to receive a highly toxic reply from the Anti-Vax group. Looking at the toxicity of Anti-Vax’s inter-cluster replies by target (see SI), it was found that the Anti-Vax’s high toxicity is significantly directed at the Right cluster in English tweets. Upon scrutinising the content of the highly toxic replies from Anti-Vax to Right, we found that most of the replies were among the lines of ‘Don’t spread the vaccine’. As the Trump administration was in power, it seems that complaints and requests were made against the administration with high aggressiveness, even though the Right cluster was close to the Anti-Vax cluster in the RT network. On the contrary, in Japanese tweets, most of the toxic replies from Anti-Vax to Right were about criticism against the administration party, although they were not concerned with the vaccine rollout. The toxicity from the Anti-Vax to Right clusters was not significantly high compared to those of other clusters.

Moreover, we found a positive correlation between toxicity and the number of followers of the target of Anti-Vax replies both in English and Japanese. Figure 4c, f illustrate the max toxicities one received from Anti-Vax users, indicating that users with many followers are more likely to receive replies with high toxicity. In English, however, there are a certain number of accounts with a small number of followers that still receive highly toxic replies, and if we view them as less tolerant of toxicity than people with larger followings, they should be careful about the toxic replies. We cannot find this tendency in Japanese tweets.

**Fig. 5:**
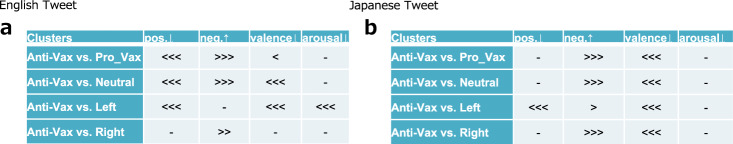
Comparison of emotions of inter-cluster replies. **a** for English and **b** for Japanese. The comparison is based on each feature using the Mann–Whitney *U*-test with a Bonferroni correction. The direction of the brackets indicates the cluster with the larger amount. ‘ < < < ’: *p* < 0.001, ‘ < < ’: *p* < 0.01, ‘ < ’: *p* < 0.05.

Finally, we evaluated the emotions of inter-cluster replies. Compared with the other clusters, replies from the Anti-Vax cluster contained more negative and fewer valence words in both English and Japanese, although the less use of positive words is apparent in English only (Fig. 5). This result is consistent with the finding that the Anti-Vax users’ replies are toxic and thus negative.

## Discussion

### Fragmentation of the vaccine information ecosystems

We have found topically and structurally distinct clusters in the vaccine information ecosystems for both English and Japanese tweets. Such political polarisation and echo chambers have been long discussed in the literature. It is known that the stronger the ideology of a group, the more isolated it is on social media (Bright, 2018). Political ideology (i.e., left-right wing) is a prime example, and existing research has observed that left and right groups tend to be divided on social media regardless of country (Bright, 2018, Ozaydin). In particular, conservatives are known to form stronger political echo chambers in the U.S. (Adamic and Glance, 2005; Boutyline and Willer, 2017; Ozaydin and Nishida, 2021). This was also confirmed by the result of this study in that the Right group was more distant from the Neutral group than the Left group in English tweets. Besides political topics, it is known that users with similar stances on vaccination preferentially interacted with each other (Mønsted and Lehmann, 2022), creating a fragmentation on social media (Cossard et al., 2020; Gunaratne et al., 2019).

Why do we see similar fragmentation in the vaccine information ecosystems? Concerning the relationship between vaccines and politics, studies revealed that conservative factions are notably correlated with anti-vaccine people and are more likely to believe vaccine conspiracy theories than liberals (Featherstone et al., 2019; Jennings et al., 2021; Muric et al., 2021). This may be because conservatives’ preference for stability (Boutyline and Willer, 2017) is consistent with the nature of anti-vaccine groups or because, as some studies have argued (Broniatowski et al., 2018; Jamison, 2020), conservatives are weaponizing the vaccine narrative, often with misinformation and anxiety-provoking statements, for their political gain. Particularly with regard to vaccines, ‘freedom of choice’ is a partisan issue rather than medical concerns, such as vaccine side effects (DiResta, 2018). For example, most conservatives place particular emphasis on individual freedom of choice in the U.S. (DiResta, 2018). Thus, the more liberals advance pro-vaccine policies, the more anti-vaccine stances become a good excuse for conservatives to denounce liberals, and consequently, anti-vaxxers gain allies who share their position. The most concern from these results is that such an echo chamber environment may cause the acceleration of political polarisation and exacerbate further social divide in the vaccine information ecosystems.

As mentioned, the vaccine information ecosystems demonstrate striking similarities in English and Japanese, although they have different vaccine policies and political backgrounds (Owen et al., 2020). For example, Japan has less political use of social media than the U.S. (Owen et al., 2020) and the right-wing has a xenophobic attitude toward China and South Korea (Fujishiro et al., 2020) rather than a conservative one (partially confirmed by this word cloud in this study) in Japan. Despite such differences, this structural and topical resemblance suggests that vaccination is a common political matter across countries (Sharun and Dhama, 2021), especially in the context of the COVID-19 pandemic.

### Aggressive reply behaviours of anti-vaxxers

We have revealed that the replies of anti-vaxxers are highly active, targeted, and toxic compared to others’ replies. Consequently, the reply of anti-vaxxers can work as a targeted attack to influence vaccine belief and may provoke anxiety about vaccination in other clusters, because replies can reach beyond a follow-follower network. These characteristics are common in English and Japanese, although Western countries and Japan have different cultures and policies about vaccination. The repeated exposure to the same belief can affect people’s perception efficiently, in what is known as the ‘mere exposure effect’ (Bornstein, 1989a), coupled with subliminal techniques often used in propaganda (Bornstein, 1989b; Bornstein and D’agostino, 1992). Furthermore, because their replies were directed to popular accounts such as news media, the possibility of being witnessed by users in other communities would be impactful. This kind of borrowing of the authority of prominent social media accounts is a typical strategy for spreading disinformation (Benkler et al., 2018; Watts et al., 2021). Thus, a potential reason the anti-vaxxers actively send replies to neutral accounts (mostly media accounts) would be to attempt to propagate anti-vaccine beliefs and strengthen their influence.

Our study also demonstrated that the inter-cluster replies of anti-vaxxers conveyed higher toxicity and negative sentiment. Previous research on effective vaccine narratives has illustrated that texts with strong emotionality were more likely to leave a greater impression on receivers than texts with a detailed description (Betsch et al., 2011). Other researchers have reported that influential users on social media tend to be individuals who express negative sentiments (Quercia et al., 2011; Xiao and Khazaei, 2019). Emotional messages effectively affect users who just witnessed the message even though they are not directed at them (Grandjean et al., 2005). Considering this evidence, the toxic attack by the Anti-Vax is a potentially dangerous behaviour that may increase vaccine hesitancy and put anti-vaccine beliefs into other groups’ minds.

Given the above characteristics of anti-vaxxers’ replies that are common across languages, two important implications arise. First, platformers will be able to take measures to avoid exposing anti-vaccine discourse to other users. Reply behaviours can jump over the follow-follower relationships, and thus they can be exposed to the recipients and the followers of the recipients. Although reply behaviour cannot be banned due to the freedom of expression, platformers can consider varying the priority of displaying replies or adding some delay, especially replies toward prominent accounts. The reply to prominent accounts is one place where we can see tweets with different beliefs. If there can be anti-vaccine-related replies, lowering the priority of displaying these replies would decrease the possibility of exposing them to other users.

Second, pro-vaxxers should prepare for toxic and emotional replies from anti-vaxxers. In the interviews with pro-vax organisations about their experiences with responding to anti-vaxxers (Steffens et al., 2019), an interviewee highlighted the ‘need to come across as the responsible, reasonable, calm ones because of all the people that are reading and not commenting’. This study also found that anti-vaxxers were negative in their expressions and tone. Those findings, including ours, should be shared by social media users and platformers. Although there are several attempts to fact-check vaccine misinformation^12^, fact-checking organisations should make more actionable guidelines to deal with high toxic replies from anti-vaxxers.

Equipped with countermeasures based on language-dependent and independent features of anti-vaxxers, such as ours, we can better guard from the toxic targeted attack by anti-vaxxers both at individual and platform levels.

## Supplementary information

Additional Info File - SUPPLEMENTAL_MATERIAL_pdf

## Data Availability

The tweet IDs used in this study is available at https://github.com/Mmichio/Aggressive_Behaviour_of_Antivaxxers_public.

## Footnotes

1 https://www.who.int/health-topics/infodemic.

2 https://en.wikipedia.org/wiki/Impression_(online_media).

3 Twitter recently added a feature that allows users limit accounts that can reply to them, but most users still permit free reply access to them. https://techcrunch.com/2020/08/11/twitter-now-lets-everyone-limit-replies-to-their-tweets/.

4 https://www.perspectiveapi.com/.

5 https://cloud.google.com/translate/.

6 Dr. Fauci (‘fauchi’), the director of the NIAID.

7 National Institute of Allergy and Infectious Diseases https://www.niaid.nih.gov/.

8 https://www.gatesfoundation.org/our-work/programs/global-health/vaccine-development-and-surveillance.

9 The Ministry of Health, Labor and Welfare of the Japanese government.

10 On Twitter, accounts with followers of more than 10,000 have much influence, and the number of 10,000 is an official milestone to be recognised as a large account https://www.cnbc.com/2021/09/01/how-to-use-twitter-super-follows-to-earn-money.html.

11 On the other hand, we have to note that there were fewer tweets with very high toxicity overall (e.g., tweets with toxicity scores above 0.7 (Hua et al., 2020a, b) or 0.5 (Gehman et al., 2020)), regardless of the cluster.

12 https://vaccinationdemandobservatory.org/.

## Code availability

The code used for analysis is available at https://github.com/Mmichio/Aggressive_Behaviour_of_Antivaxxers_public. The open-source software Gephi and Python libraries were used to compute statistics and produce the figures.

## References

[CR1] Adamic LA, Glance N (2005) The political blogosphere and the 2004 us election: divided they blog. In: Proceedings of the 3rd international workshop on Link discovery. Association for Computing Machinery, pp. 36–43

[CR2] Agley J, Xiao Y (2021). Misinformation about covid-19: evidence for differential latent profiles and a strong association with trust in science. BMC Public Health.

[CR3] Alvarez-Hamelin JI, Dall’Asta L, Barrat A, Vespignani A (2006) Large scale networks fingerprinting and visualization using the k-core decomposition. Adv Neural Inform Process Syst 18, pp 41–50

[CR4] Benkler Y, Faris R, Roberts H (2018) Network Propaganda: Manipulation, Disinformation, and Radicalization in American Politics. Oxford University Press

[CR5] Betsch C, Ulshöfer C, Renkewitz F, Betsch T (2011). The influence of narrative v. statistical information on perceiving vaccination risks. Med Decis Making.

[CR6] Blondel VD, Guillaume Jean-Loup, Lambiotte R, Lefebvre E (2008). Fast unfolding of communities in large networks. J Stat Mechan: Theory Exp.

[CR7] Bornstein RF (1989). Subliminal techniques as propaganda tools: review and critique. J Mind Behav.

[CR8] Bornstein RF (1989). Exposure and affect: overview and meta-analysis of research, 1968–1987. Psychol Bull.

[CR9] Bornstein RF, D’agostino PR (1992). Stimulus recognition and the mere exposure effect. J Person Soc Psychol.

[CR10] Boutyline A, Willer R (2017). The social structure of political echo chambers: Variation in ideological homophily in online networks. Polit Psychol.

[CR11] Brennen JS, Simon F, Howard PN, Nielsen RasmusKleis (2020). Types, sources, and claims of covid-19 misinformation. Reuters Institute.

[CR12] Bright J (2018). Explaining the emergence of political fragmentation on social media: The role of ideology and extremism. J Comput-Mediat Commun.

[CR13] Broniatowski DA, Jamison AM, Qi SiHua, AlKulaib L, Chen T, Benton A, Quinn SC, Dredze M (2018). Weaponized health communication: Twitter bots and russian trolls amplify the vaccine debate. Am J Public Health.

[CR14] Burki T (2019). Vaccine misinformation and social media. Lancet Digi Health.

[CR15] Choi D, Chun S, Oh H, Han J (2020). Rumor propagation is amplified by echo chambers in social media. Sci Rep.

[CR16] Conover M, Ratkiewicz J, Francisco M, Gonçalves B, Menczer F, Flammini A (2011) Political polarization on twitter. In: Proceedings of the International AAAI Conference on Web and Social Media, vol. 5, 89-96, AAAI Press

[CR17] Cossard A, De Francisci Morales G, Kalimeri K, Mejova Y, Paolotti D, Starnini M (2020) Falling into the echo chamber: the Italian vaccination debate on twitter. In: Proceedings of the International AAAI Conference on Web and Social Media, vol, 14. pp. 130–140, AAAI Press,

[CR18] Destiny Apuke O, Omar B (2021). Fake news and covid-19: modelling the predictors of fake news sharing among social media users. Telemat Inform.

[CR19] DiResta R (2018) Of virality and viruses: the anti-vaccine movement and social media. NAPSNet Special Reports, vol. 8, Nautilus Institute for Security and Sustainability

[CR20] Featherstone JD, Bell RA, Ruiz JB (2019). Relationship of people’s sources of health information and political ideology with acceptance of conspiratorial beliefs about vaccines. Vaccine.

[CR21] Fontanet A, Cauchemez S (2020). Covid-19 herd immunity: where are we?. Nat Rev Immunol.

[CR22] Fortunato S (2010). Community detection in graphs. Phys Rep.

[CR23] Fujishiro H, Mimizuka K, Saito M (2020) Why doesn’t fact-checking work? The mis-framing of division on social media in Japan. In: International Conference on Social Media and Society, pp. 309–317, Association for Computing Machinery

[CR24] Garimella K, De Francisci Morales G, Gionis A, Mathioudakis M (2018). Quantifying controversy on social media. ACM Trans Soc Comput.

[CR25] Gehman S, Gururangan S, Sap M, Choi Y, Smith NA (2020) Realtoxicityprompts: evaluating neural toxic degeneration in language models. In: Findings of the Association for Computational Linguistics: EMNLP 2020. Association for Computational Linguistics. pp. 3356–3369

[CR26] Germani F, Biller-Andorno N (2021). The anti-vaccination infodemic on social media: a behavioral analysis. PLoS ONE.

[CR27] Grandjean D, Sander D, Pourtois G, Schwartz S, Seghier ML, Scherer KR, Vuilleumier P (2005). The voices of wrath: brain responses to angry prosody in meaningless speech. Nat Neurosci.

[CR28] Gunaratne K, Coomes EA, Haghbayan H (2019). Temporal trends in anti-vaccine discourse on twitter. Vaccine.

[CR29] Hua Y, Naaman M, Ristenpart T (2020a) Characterizing twitter users who engage in adversarial interactions against political candidates. In: Proceedings of the 2020 CHI Conference on Human Factors in Computing Systems. pp. 1–13, Association for Computing Machinery

[CR30] Hua Y, Ristenpart T, Naaman M (2020b) Towards measuring adversarial twitter interactions against candidates in the us midterm elections. In: Proceedings of the International AAAI Conference on Web and Social Media, vol. 14, pp. 272–282, AAAI Press

[CR31] Jamison AM (2020). Vaccine communication as weaponized identity politics. Am J Public Health.

[CR32] Jennings W, Stoker G, Bunting H, Valgardsson ViktorOrri, Gaskell J, Devine D, McKay L, Mills MC (2021). Lack of trust, conspiracy beliefs, and social media use predict covid-19 vaccine hesitancy. Vaccines.

[CR33] Johnson NF, Velásquez N, Restrepo NicholasJohnson, Leahy R, Gabriel N, El Oud S, Zheng M, Manrique P, Wuchty S, Lupu Y (2020). The online competition between pro-and anti-vaccination views. Nature.

[CR34] Kata A (2012). Anti-vaccine activists, web 2.0, and the postmodern paradigm–an overview of tactics and tropes used online by the anti-vaccination movement. Vaccine.

[CR35] Mitra T, Counts S, Pennebaker J (2016) Understanding anti-vaccination attitudes in social media. In: Proceedings of the International AAAI Conference on Web and Social Media, vol. 10, AAAI Press

[CR36] Mønsted B, Lehmann S (2022). Characterizing polarization in online vaccine discourse-a large-scale study. PLoS ONE.

[CR37] Muric G, Wu Y, Ferrara E (2021). Covid-19 vaccine hesitancy on social media: Building a public twitter data set of antivaccine content, vaccine misinformation, and conspiracies. JMIR Public Health Surveill.

[CR38] Owen D, Ogasahara M, Kiyohara S (2020) Public perceptions of “fake news” in the United States and Japan, APSA Preprints, Cambridge University Press

[CR39] Pennebaker JW, Boyd RL, Jordan K, Blackburn K (2015) The development and psychometric properties of liwc2015. Technical report, Texas Scholar Works

[CR40] Quercia D, Ellis J, Capra L, Crowcroft J (2011) In the mood for being influential on twitter. In: 2011 IEEE Third International Conference on Privacy, Security, Risk and Trust and 2011 IEEE Third International Conference on Social Computing. IEEE. pp. 307–314

[CR41] Samoilenko A, Lemmerich F, Weller K, Zens M, Strohmaier M (2017) Analysing timelines of national histories across wikipedia editions: A comparative computational approach. In: Proceedings of the International AAAI Conference on Web and Social Media, vol. 11, AAAI Press

[CR42] Sasahara K, Chen W, Peng H, Ciampaglia GL, Flammini A, Menczer F (2020). Social influence and unfollowing accelerate the emergence of echo chambers.. J Comput Soc Sci.

[CR43] Sharun K, Dhama K (2021) Covid-19 vaccine diplomacy and equitable access to vaccines amid ongoing pandemic. Archives of medical research vol. 52(7): 761−763, Elsevier 10.1016/j.arcmed.2021.04.006 PMC8062433 33941393

[CR44] Steffens MS, Dunn AG, Wiley KE, Leask J (2019). How organisations promoting vaccination respond to misinformation on social media: a qualitative investigation. BMC Public Health.

[CR45] Warriner AB, Kuperman V, Brysbaert M (2013). Norms of valence, arousal, and dominance for 13,915 english lemmas. Behav Res Method.

[CR46] Watts DJ, Rothschild DM, Mobius M (2021). Measuring the news and its impact on democracy. Proc Natl Acad Sci USA.

[CR47] Wu S, Resnick P (2021) Cross-partisan discussions on youtube: conservatives talk to liberals but liberals don’t talk to conservatives. https://arxiv.org/abs/2104.05365

[CR48] Xiao L, Khazaei T (2019) Changing others’ beliefs online: Online comments’ persuasiveness. In: Proceedings of the 10th International Conference on Social Media and Society. pp. 92–101, Association for Computing Machinery

[CR49] Yurtcicek Ozaydin S, Nishida R (2021). Fragmentation and dynamics of echo chambers of turkish political youth groups on twitter. J Socio-Inform.

[CR50] Yurtcicek Ozaydin S (2021) Hashtag wars, online political polarization and mayoral elections. Sosyal Bilimler Araştırma Dergisi 10(3):1–10

