# Supplementary material for: Aggressive behaviour of anti-vaxxers and their toxic replies in English and Japanese
Source: Humanit Soc Sci Commun. 2022 Jul 5;9(1):229. doi: 10.1057/s41599-022-01245-x (PMC9255452; doi:10.1057/s41599-022-01245-x)
Supplement: Supplementary file 1 — Additional Info File - SUPPLEMENTAL_MATERIAL_pdf [file 41599_2022_1245_MOESM1_ESM.pdf]

Supplementary Material

Aggressive Behaviour of Anti-vaxxers and Their Toxic Replies in English and Japanese

Kunihiro Miyazaki[1*], Takayuki Uchiba[2], Kenji Tanaka[1] and Kazutoshi Sasahara[3*]

[1]The University of Tokyo, Japan.
[2]Sugakubunka Co., Ltd., Japan.
[3]Tokyo Institute of Technology, Japan.

*Corresponding author(s). E-mail(s): kunihirom@acm.org; sasahara.k.aa@m.titech.ac.jp;
Contributing authors: takayuki.uchiba@sugakubunka.com; tanaka@tmi.t.u-tokyo.ac.jp;

English tweets

a Pro-Vax

#	Screen Name	Description
1	kylegriffin1	Media Producer
2	funder	Journalist, Founder of anti-Trump org.
3	atrupar	journalist
4	DrDenaGrayson	MD, Ebola expert
6	eugenegu	Editor

b Anti-Vax

#	Screen Name	Description
1	davidkurten	British politicial, conservative
6	HighWireTalk	Webcast of a specific producer
7	ChildrensHD	Anti-Vax activist group
8	johncardillo	Former New York City Police
9	Mike_Pence	Ex-Vice President

c Neutral

#	Screen Name	Description
1	Reuters	News Media
2	nytimes	News Media
3	CNN	News Media
4	spectatorindex	News Media
5	SkyNews	News Media

d Right

#	Screen Name	Description
1	WhiteHouse	The White House
2	RealCandaceO	Author, Republican
3	Thomas1774Paine	Journalist
6	disclosetv	online community
7	21WIRE	journalist

e Left

#	Screen Name	Description
1	DrEricDing	Epidemiologist, health economist
2	HelenBranswell	Writer
3	Laurie_Garrett	Author
4	WHO	World Health Organization
5	Craig_A_Spencer	MD, Ebola Survivor

Japanese tweets

f Pro-Vax

#	Screen Name	Description
1	io302	MD
2	mph_for_doctors	MD
3	EARL_Med_Tw	MD, infectious disease expert
4	VaccineWatch	Biotechnology scientist
5	sekkai	MD

g Anti-Vax

#	Screen Name	Description
1	nakamuraclinic8	MD
2	JunSakura_Japan	information spreader
3	TTrumpSJapan	information spreader
4	someone5963	information spreader
5	BABYLONBU5TER	Worker at daycare center

h Neutral

#	Screen Name	Description
1	ReutersJapan	News Media
2	nhk_news	News Media
3	YahooNewsTopics	News Media
4	nikkei	News Media
5	livedoornews	News Media

i Right

#	Screen Name	Description
1	SatoMasahisa	member of the House of Councillors
2	sonkoubun	Chinese Manga Writer
3	anonymous_post2	News summary website
4	dappi2019	information spreader
5	Tomo20309138	information spreader

j Left

#	Screen Name	Description
1	BB45_Colorado	Writer
2	influenzer3	MD
3	masahirono	Immunologist
4	GoodBye_Nuclear	MD
5	keyaki1117	Ex-Member of the House of Representatives

Fig. 1 Most retweeted accounts in Pro-Vax (a), Anti-Vax (b), Neutral (c), Right (d) and Left (e), and Japanese counterparts (f, g, h, i, j). Each table shows the screen names and description of accounts based on their bio infomation.

a

English tweets

Cluster	Most retweeted tweets
Pro-Vax	More than 90 vaccines are being developed against SARS-CoV-2 across the world. At least six groups have begun injecting formulations into volunteers in safety trials. Here is a graphical guide explaining each vaccine design. #COVID19
Anti-Vax	Bill Gates' plan to "save the world" by rolling out a potentially mandatory vaccine for the coronavirus - & suggesting nobody will be allowed to resume "normal life" without a "digital certificate" of vaccination - is proving extremely unpopular among people who follow logic.
Neutral	Coronavirus vaccine developed by Oxford University appears safe and trains the immune system, key early trials show
Right	EXCLUSIVE: Robert F. Kennedy Jr. Drops New Bombshell - Bill Gates' Coronavirus Vaccine Will Pay Out BILLIONS in Profits to Dr. Fauci's Agency
Left	The Biden-Harris plan to get coronavirus under control includes: free COVID-19 testing, treatment, and vaccines for everyone.

b

Japanese tweets

Cluster	Most retweeted tweets (Translated)
Pro-Vax	If you search for "new coronavirus", you will get pessimistic news, but if you search for the official disease name "COVID-19", you will get calm information, and if you search for the virus name "SARS-CoV-2", you will get calm information. Search for the latest results in vaccines and remedies. The amount of anxiety may be with the antenna.
Anti-Vax	FDA cancels Bill Gates coronavirus test program<br>Vaccines with microchips are likely to be avoided in the United States.<br>Many Japanese politicians wear SDGs badges, and the media is pushing Gates, so it is important to awaken the people to prevent it.<br>The SDGs Chip Program is not a conspiracy theory.
Neutral	It seems to be exactly what I had repeatedly predicted, "It is impossible to realize a new corona vaccine that can be a permanent silver bullet."
Right	Today, a member of the Liberal Democratic Party said, "You've been making radical remarks about the new coronavirus."<br>However, since there are no remedies or vaccines for new types of infectious diseases, it may be irreversible unless we take the initiative in crisis management. Even if it is called Taliban Sato, I will continue to insist on what is necessary.
Left	Can you stop doing this anymore? This time it's life-threatening.<br>New corona, vaccine production ignored by Ministry of Health, Labor and Welfare Amakudari overseas products (news post seven)

Fig. 2 Most retweeted tweets in Pro-Vax (a), Anti-Vax (b), Neutral (c), Right (d) and Left (e), and Japanese counterparts (f, g, h, i, j). Japanese tweets are translated by Google Translate API.

a English Tweet

		Follower Counts		
		Target	Source	All
Reply Source	Pro-Vax	234,687	1,863	995
	Left	1,318,926	2,254	885
	Anti-Vax	455,353	699	400
	Right	218,730	2,913	1,241
	Neutral	258,386	1,601	797

b Japanese Tweet

		Follower Counts		
		Target	Source	All
Reply Source	Pro-Vax	241,353	1,234	456
	Left	281,268	1,209	551
	Anti-Vax	469,924	567	229
	Right	469,480	1,095	522
	Neutral	73,188	634	427

Fig. 3 **Follower counts in English tweets (a) and Japanese tweets (b).** . The numbers in tables indicate the median number of followers of reply targets (Target), reply source (Source), and all accounts of each cluster (All), e.g., the median follower counts are 234,687 for Pro-Vax's reply targets, 1,863 for Pro-Vax's reply senders, and 995 for the all Pro-Vax accounts. Here, Target has much more numbers than Source and All. The median follower counts of Targets is more than 200,000 in all clusters, which is also much higher than the average number of followers in Twitter as a whole (about 1,000 according to https://kickfactory.com/blog/average-twitter-followers-updated-2016/)

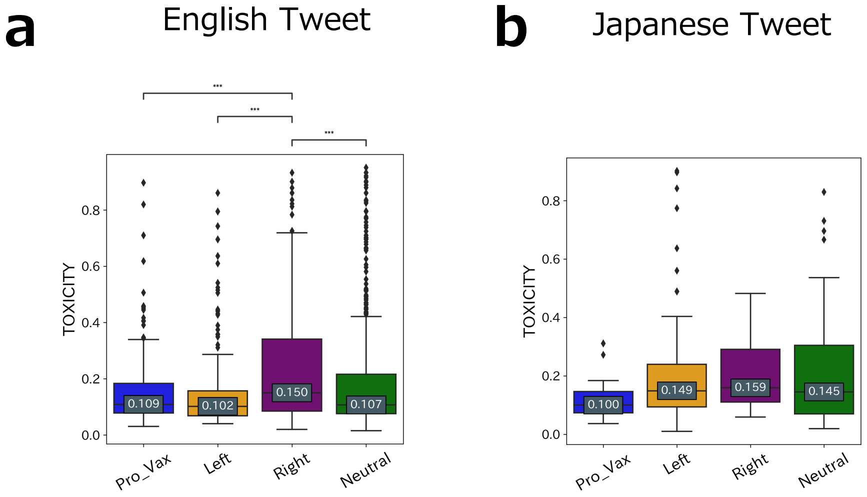

Fig. 4 **Breakdown of the toxicity score of Anti-Vax's inter-cluster replies by target cluster in English tweets (a) and Japanese tweets (b).** . The toxicity from Anti-Vax cluster to Right was higher in both languages although we could not get significance from Japanese tweets.
